# HPLC-MS-MS quantification of short-chain fatty acids actively secreted by probiotic strains

**DOI:** 10.3389/fmicb.2023.1124144

**Published:** 2023-03-03

**Authors:** Marco Calvigioni, Andrea Bertolini, Simone Codini, Diletta Mazzantini, Adelaide Panattoni, Mariacristina Massimino, Francesco Celandroni, Riccardo Zucchi, Alessandro Saba, Emilia Ghelardi

**Affiliations:** ^1^Department of Translational Research and New Technologies in Medicine and Surgery, University of Pisa, Pisa, Italy; ^2^Department of Surgical, Medical and Molecular Pathology and Critical Care Medicine, University of Pisa, Pisa, Italy; ^3^Research Center Nutraceuticals and Food for Health–Nutrafood, University of Pisa, Pisa, Italy

**Keywords:** probiotics, short-chain fatty acids, acetic acid, propionic acid, butyric acid, secretion, HPLC-MS-MS, SCFAs

## Abstract

**Introduction:**

Short-chain fatty acids (SCFAs) are the main by-products of microbial fermentations occurring in the human intestine and are directly involved in the host’s physiological balance. As impaired gut concentrations of acetic, propionic, and butyric acids are often associated with systemic disorders, the administration of SCFA-producing microorganisms has been suggested as attractive approach to solve symptoms related to SCFA deficiency.

**Methods:**

In this research, nine probiotic strains (*Bacillus clausii* NR, OC, SIN, and T, *Bacillus coagulans* ATCC 7050, *Bifidobacterium breve* DSM 16604, *Limosilactobacillus reuteri* DSM 17938, *Lacticaseibacillus rhamnosus* ATCC 53103, and *Saccharomyces boulardii* CNCM I-745) commonly included in commercial formulations were tested for their ability to secrete SCFAs by using an improved protocol in high-performance liquid chromatography coupled to tandem mass spectrometry (HPLC-MS-MS).

**Results:**

The developed method was highly sensitive and specific, showing excellent limits of detection and quantification of secreted SCFAs. All tested microorganisms were shown to secrete acetic acid, with only *B. clausii* and *S. boulardii* additionally able to produce propionic and butyric acids. Quantitative differences in the secretion of SCFAs were also evidenced.

**Discussion:**

The experimental approach described in this study may contribute to the characterization of probiotics as SCFA-producing organisms, a crucial stage toward their application to improve SCFA deficiency.

## Introduction

The human gut microbiota actively cooperates in maintaining physiological balance, in nutrient catabolism and absorption, as well as in the production of short-chain fatty acids (SCFAs) (LeBlanc et al., 2017). SCFAs are small carboxylic organic acids with a backbone of at most six carbon atoms. They represent the largest group of metabolic products obtained by microbial fermentation of dietary complex carbohydrates, otherwise undegradable by the human digestive system (Oliphant and Allen-Vercoe, 2019). *Faecalibacterium prausnitzii*, *Eubacterium rectale*, *Akkermansia muciniphila*, *Clostridium* spp., *Bifidobacterium* spp., *Lactobacillus* spp., and members belonging to families *Ruminococcaceae*, *Lachnospiraceae*, and *Bacteroidaceae* are the main actors able to produce SCFAs in the human gut as result of bacterial metabolism (Markowiak-Kopeć and Śliżewska, 2020).

The highest intestinal SCFA concentrations are reached by acetic, propionic, and butyric acids, which are known to exert countless beneficial effects and orchestrate several physiological functions (Morrison and Preston, 2016; Silva et al., 2020). Acetic acid is the structurally simplest fatty acid and can, therefore, be used as metabolic substrate in fatty acid biosynthesis and Krebs’ cycle (Okamoto et al., 2019). Besides being a major energy source for skeletal muscle (Okamoto et al., 2019), acetic acid is also involved in lipid metabolism of the liver and adipose tissue (Liu et al., 2019). The administration of acetate was reported to decrease food intake, body weight gain, blood cholesterol, and triglyceride levels (Liu et al., 2019). Propionic acid improves the barrier function and epithelial integrity in the gut, and impacts on glucose and lipid liver homeostasis (Langfeld et al., 2021). Previous studies reported the ability of propionic acid to indirectly modulate gene expression and cell metabolism through immunomodulatory mechanisms (Tan et al., 2014; Langfeld et al., 2021). Butyric acid is probably the most multifunctional SCFA, acting as energy source for colonic epithelial cells and promoting beta oxidation rather than glycolysis (Riviere et al., 2016). Enhancing the expression of tight-junction proteins, butyric acid facilitates the maintenance of the intestinal barrier integrity, thus hampering epithelial invasion by pathogenic microorganisms (Riviere et al., 2016). It has also been revealed to inhibit tumoral cell expansion and release of pro-inflammatory cytokines (Liu et al., 2021; Gheorghe et al., 2022), thus demonstrating anti-carcinogenic and anti-inflammatory properties. All the main SCFAs were found able to differently modulate appetite and energy intake by increasing colonic glucagon-like peptide-1 secretion (Christiansen et al., 2018), thus reducing food craving (Goswami et al., 2018). Moreover, they are also directly involved in the microbiota-gut-brain axis neuroendocrine signaling, brain physiology, and neuronal damage prevention (O’Riordan et al., 2022).

A correlation between lower intestinal SCFA concentrations and the exacerbation of different pathological conditions, such as inflammatory bowel diseases and neurological and neuropsychiatric disorders (e.g., autism, depression, Alzheimer and Parkinson’s diseases, multiple sclerosis), has been confirmed (Sun et al., 2017; Kong et al., 2018; Blaak et al., 2020; Tobin et al., 2021; O’Riordan et al., 2022). The administration of properly selected probiotic microorganisms able to counteract SCFA deficiency appeared as a promising novel approach to co-adjuvate the management of these conditions in humans and improve pathology-associated symptoms. For instance, psychobiotics producing SCFAs, neurotransmitters, and neuroendocrine hormones were revealed to provide wide health benefits to patients suffering from mental and neurological disorders (Cheng et al., 2021), thus pointing out SCFA production by probiotics as an additional beneficial feature in certain clinical conditions. Several studies demonstrated the ability of some probiotics to shift the gut microbiota composition toward higher abundances of SCFA-producing species (Markowiak-Kopeć and Śliżewska, 2020). However, probiotic strains themselves were scarcely tested for the direct production of SCFAs. Studies focusing on this aspect demonstrated that probiotic microorganisms were able to secrete acetic, propionic, and butyric acids in different amounts and modalities often dependent on the tested species or strain (Asarat et al., 2015; Kahouli et al., 2015; LeBlanc et al., 2017; Cheng et al., 2021). For this reason, an in-depth characterization of commercialized probiotics as concern SCFA production should be performed for an optimal targeted bacteriotherapy.

In this study, nine probiotic strains included in commercial formulations worldwide were tested for their ability to actively secrete acetic acid, propionic acid, and butyric acid and the amount of secreted SCFAs was quantified by using an optimized and sensitive protocol in high-performance liquid chromatography coupled to tandem mass spectrometry (HPLC-MS-MS).

## Materials and methods

### Materials and chemicals

Acetic acid (purity ≥99.8%), propionic acid (≥99.5%), propionic acid-2,3-^13^C_2_ (99 atom% ^13^C), and butyric acid (≥99.5%) analytical standards, water LC-MS grade, acetonitrile (ACN) LC-MS grade, formic acid (FA, ≧ 98%), hydrochloric acid (ACS reagent, 37% w/w), NaOH solution 1 M, diethyl-ether (≥99%), 3-nitrophenylhydrazine (3NPH) hydrochloride, N-(3-dimethylaminopropyl)-N′-ethyl carbodiimide (EDC) hydrochloride, Bifidus selective medium (BSM), BSM supplement, and Sabouraud-2% dextrose agar (SDA) were bought from Merck KGaA (Darmstadt, Germany). Acetic acid ^13^C_2_ (99 atom% ^13^C) analytic standard was purchased from Cambridge Isotope Laboratories (Tewksbury, MA, USA). Brain Heart Infusion (BHI) agar was obtained from Biolife (Monza, Italy), while Trypticase Soy agar supplemented with 5% horse blood (TSH) was from bioMérieux (Paris, France). De Man, Rogosa, and Sharpe (MRS) agar was acquired from Thermo Fisher Scientific (Waltham, MA, USA).

### Microbial strains and growth conditions

Nine microbial strains contained in worldwide commercialized, mono-species probiotic formulations were selected and tested in the present investigation. In particular, *Bacillus clausii* NR, OC, SIN, and T (isolated from Enterogermina, Sanofi, Paris, France, and declared on the product label), *Bacillus coagulans* ATCC 7050 (isolated from Lactò Più, Recordati, Milan, Italy, and declared on the product label), *Bifidobacterium breve* DSM 16604 (isolated from Neovaxitiol, IBSA Farmaceutici, Lodi, Italy, and declared on the product label), *Limosilactobacillus reuteri* DSM 17938 (isolated from Reuflor, Italchimici, Brescia, Italy, and declared on the product label with the old nomenclature *Lactobacillus reuteri* DSM 17938), *Lacticaseibacillus rhamnosus* ATCC 53103 (isolated from Dicoflor, Dicofarm, Roma, Italy, and declared on the product label with the old nomenclature *Lactobacillus rhamnosus* ATCC 53103), and *Saccharomyces boulardii* CNCM I-745 (isolated from Codex, Zambon, Bresso, Italy, and declared on the product label) were tested. All probiotic products were purchased from local pharmacies. *B. clausii* NR, OC, SIN, and T were isolated from Enterogermina as described in a previous work (Senesi et al., 2001). *B. coagulans* was seeded on TSH and plates were incubated at 37°C for 48 h. *B. breve* was propagated on BSM containing 0.116 g/L of BSM supplement and grown at 37°C for 48–72 h in anaerobic atmosphere, generated by using Thermo Scientific™ AnaeroGen™ Compact (Thermo Fisher Scientific, Waltham, MA, USA). *L. reuteri* and *L. rhamnosus* were streaked on MRS agar and plates were incubated at 37°C for 48 h in 5% CO_2_-enriched atmosphere, generated by using Thermo Scientific™ CO_2_Gen™ Compact (Thermo Fisher Scientific, Waltham, MA, USA). *S. boulardii* was seeded on Sabouraud-2% dextrose agar and plates were incubated at 30°C for 48 h.

### Preparation of culture supernatants

Different liquid culture media (i.e., Luria Bertani broth, BHI and BHIG broths, RPMI 1640 medium) were firstly tested for microbial propagation. BHIG broth was selected as the most suitable culture medium in the end, as it allowed an optimal uniform replication of all microorganisms and contained amino acids and glucose, which are important substrates for SCFA biosynthesis (Louis and Flint, 2017). Thus, for each strain, a well-isolated colony was inoculated in 5 mL of BHIG broth. Suspensions were incubated overnight at 37°C. While *B. clausii* strains, *B. coagulans*, and *S. boulardii* were aerobically incubated, *B. breve* was incubated in anaerobic atmosphere and *L. reuteri* and *L. rhamnosus* in 5% CO_2_-enriched atmosphere. Subsequently, 100 μL of microbial cultures were inoculated in 25 mL of fresh BHIG medium. Cultures were incubated at 37°C up to an optical density at 600 nm (OD_600_) of 1.8 and then centrifuged at 3,870 rcf for 20 min at 4°C. Supernatants were collected and filtered using 0.22 μm filters to completely remove microbial cells. Supernatants were produced three times in separate days and stored at −80°C until use.

### Sample preparation

Microbial supernatants underwent a liquid-liquid extraction procedure before HPLC-MS-MS analysis, as previously described by De Baere et al. (2013) with protocol modifications. Briefly, 200 μL of each sample were placed in a 2 mL tube and added with 10 μL of a 10 μg/mL internal standard mixture made of ^13^C_2_-acetic acid and ^13^C_2_-propionic acid. The former was used as the internal standard (IS) for the quantification of acetic acid, while the latter for both propionic and butyric acids. Samples were mixed and equilibrated at room temperature for 5 min. Thereafter, 20 μL of HCl 37% w/w were added, samples were mixed for 15 sec, and extracted for 20 min by gently shaking in an orbital shaker, using 1 mL of diethyl-ether. After a centrifugation step of 5 min at 1,230 rcf, the organic phase was transferred to new tubes and 100 μL of NaOH 1 M were added. Samples were shaked again for 20 min and then centrifuged. The organic phase was removed and the aqueous phase containing SCFAs was added with 10 μL of HCl 37% in order to obtain a pH value in the range 4–7. Actually, pH is one of the limiting conditions of the derivatization process that was carried out to change the analytes’ structure, thus improving chromatographic separation and enhancing instrumental sensitivity. It can be achieved as follows: 50 μL of each sample was added with 50 μL of HPLC-MS water and then derivatization was performed adding 50 μL of 3-nitrophenylhydrazine (3NPH) hydrochloride 200 mM and 50 μL of N-(3-dimethylaminopropyl)-N′-ethyl carbodiimide hydrochloride 120 mM. 3NPH was employed to convert SCFAs to their 3-nitrophenylhydrazine form, which had showed an excellent in-solution chemical stability (Han et al., 2015). Solutions were incubated at 40°C for 30 min in constant shaking. Afterward, the derivatization reaction was quenched adding 200 μL of 0.1% formic acid, and derivatized samples were ready to be injected into the HPLC-MS-MS system for analysis. Quantification made use of calibration curves, prepared by serial dilution with water of stock standard solutions at the concentration of 1 μg/mL (for propionic and butyric acids) and 5 μg/mL (for acetic acid), to obtain concentrations of 1,000, 500, 250, 125, 62.5, 31.25, 15.63, 7.81, 3.90, and 1.95 ng/mL for acetic acid, and 200, 100, 50, 25, 12.5, 6.25, 3.13, 1.56, 0.78, and 0.39 ng/mL for propionic and butyric acids. Each calibration point (50 μL) was diluted with 50 μL of sterile BHIG, previously extracted according to the procedure used for samples, and added with a proportional amount of the IS mixture in order to achieve, in all calibrators, the same initial concentration of IS in the samples. The use of the sample matrix to build the calibration curves makes calibrators similar to the samples, providing more reliable and reproducible results. Derivatization and injection of calibration points were then performed as described above.

### HPLC-MS-MS analysis

The instrumental layout consisted in a 1,290 ultra high performance liquid chromatography (UHPLC) Infinity II system (Agilent, Santa Clara, CA, USA), including a binary pump, a column oven set at 40°C, and a thermostated autosampler, coupled to a QTRAP 6500 + LC-MS-MS (Sciex, Concord, ON, Canada), working as a triple quadrupole and equipped with an IonDrive™ Turbo V source (Sciex). Chromatographic separation was achieved by using a 110 Å, 2 × 50 mm, 3 μm particle size, Gemini C18 HPLC column (Phenomenex, Torrance, CA, USA) protected by a C18 SecurityGuard™ cartridge (Phenomenex) and using acetonitrile containing 0.1% formic acid (solvent A, A) and water with 0.1% formic acid (solvent B, B) as mobile phases. Gradient elution, with a 500 μL/min flow rate, was performed as follows: 0.0–0.3 min (A) 10%, 2.5–3.5 min (A) 20%, 3.6–4.5 min (A) 90%, 4.6–5.5 (A) 10%. Injection volume was set at 5 μL. System control, data acquisition, and data processing were performed using Sciex Analyst^®^ software (version 1.7.2). A mass spectrometry selected reaction monitoring method was operated in negative ion mode. For each compound, after the optimization of declustering potential (DP, −80 V), collision energy, and collision exit potential (Supplementary Table 1), three transitions were considered in the analysis. Based on the highest signal/noise ratios, one of them was used as quantifier (Q) and the other two as qualifiers (q) (Supplementary Table 1). Further operative parameters were set as follows: gas source 1, 45 arbitrary units; gas source 2, 30 arbitrary units; ion spray voltage, −4.5 kV; source temperature, 500°C; curtain gas, 35 arbitrary units; collision gas, N_2_; operative pressure with collision gas on, 3 mPa; entrance potential, −10 V.

### Method validation

To evaluate the method ability to differentiate the molecules of interest from other possible components and interferents present in samples, specificity was checked by repeated injections of analytes into the system and their retention times were monitored. Linearity was evaluated within the calibration curve range built as aforementioned, while instrumental sensitivity was assessed by evaluating limits of analyte detection (LOD) and quantification (LOQ). Using the S-to-N Script tool of Sciex Analyst^®^ software, concentrations providing a S/N ratio close to 3 and 10 were assumed as LOD and LOQ, respectively. Recovery and matrix effect were calculated as reported by Matuszewski et al. (2003). Recovery was evaluated by comparing the peak areas of analytes added to blank BHIG broth before and after the extraction procedure, while the estimation of matrix effect was performed comparing the peak areas of the analytes added to water (A) and blank BHIG broth (B) previously subjected to the extraction process [(B/A) × 100]. Precision (%), expressed as relative standard deviation (RSD%), intra-day and inter-day accuracies, calculated with the formula [(measured concentration/nominal concentration spiked) × 100], were measured in blank samples spiked with three different concentration levels of analytes (5 ng/mL, 50 ng/mL, and 250 ng/mL for acetic acid, 1 ng/mL, 10 ng/mL, and 50 ng/mL for propionic and butyric acids). Stability of analytes as a result of a freeze-thaw cycle was evaluated, as well. Aliquots of freshly prepared samples, spiked at low, medium, and high concentrations (already used for the calculation of accuracies) were immediately injected and results were compared to those from a second aliquot of the same concentration frozen at −20°C and thawed at room temperature before the assay.

### Statistical analysis

Data are expressed as the mean ± standard deviation. For each strain, three biological replicates with three technical replicates each were performed. All statistical analyses were performed with GraphPad Prism 8 (GraphPad Software Inc., USA). To separately infer statistically significant differences in the production of acetic, propionic, and butyric acids by probiotic microbes, the one-way ANOVA followed by Tukey’s test for multiple comparisons was applied for comparing the mean amount of SCFAs produced by each strain. Statistical significance was set at a *P*-value of < 0.05.

## Results

### Method validation

**FIGURE 1:**
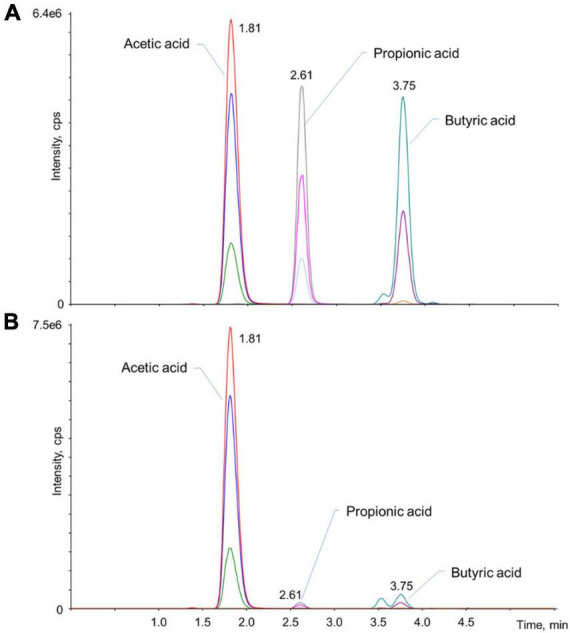
Representative chromatograms of the analytes in a standard mixture (1 μg/mL acetic acid, 200 ng/mL propionic, and butyric acids) **(A)** and in one of the real samples **(B)**. For each analyte, three multiple reaction monitoring (MRM) transitions were monitored: the one with the higher signal/noise ratio, which usually correspond to the most intense trace, was used for the quantification of the analyte (Q), while the other two transitions confirmed that the peak is attributable to the analyte (q). All MRM transitions are summarized in Supplementary Table 1.

Traces and specific retention times of acetic, propionic, and butyric acids from standard solutions and samples were achieved as shown in Figure 1. Each compound exhibited three traces, each corresponding to a specific multiple reaction monitoring (MRM) transition: the one possessing the higher signal to noise ratio was used for quantification (quantifier, Q) of the analyte, while the others, which are representative of the specific analyte structure, confirmed its identity (qualifier, q). Retention time further confirmed the peak correspondence. These features demonstrated excellent specificity and sensitivity (LOD and LOQ values) of the HPLC-MS-MS method for the quantification of SCFAs. Their values are reported in Table 1 together with the recovery of the extraction process and contribution of matrix effect. Linearity resulted ≥0.9997 for each analyte. Methodological inter-day and intra-day accuracy was in the optimal range of 85–115%, which is in compliance with the European Medicines Agency guidelines (European Medicines Agency, 2015). Precision, representing the closeness among repeated individual measurements of the analytes, was always < 7%. Re-analysis of samples after storage showed no degradation of analytes or their relative internal standards, thus confirming the stability of our protocol.

**TABLE 1:** Retention times, LODs and LOQs, recovery, and matrix effect rates for acetic, propionic, and butyric acids, separately.

Analyte	Retention time (min)	LOD/LOQ (pg/mL)	Recovery (%)	Matrix effect (%)
Acetic acid	1.82 ± 0.01	4.90/9.80	91.93 ± 9.02	91.17 ± 7.85
Propionic acid	2.62 ± 0.02	1.95/3.90	117.75 ± 13.20	95.96 ± 14.49
Butyric acid	3.76 ± 0.01	1.95/3.90	104.40 ± 4.78	117.25 ± 3.89

### Quantification of the SCFA amount in culture supernatants

*Bacillus clausii*, *B. coagulans*, *B. breve*, *L. reuteri*, *L. rhamnosus*, and *S. boulardii* supernatants collected from actively replicating cells were subjected to a HPLC-MS-MS analysis to determine the amount of acetic, propionic, and butyric acids secreted in the culture medium.

**FIGURE 2:**
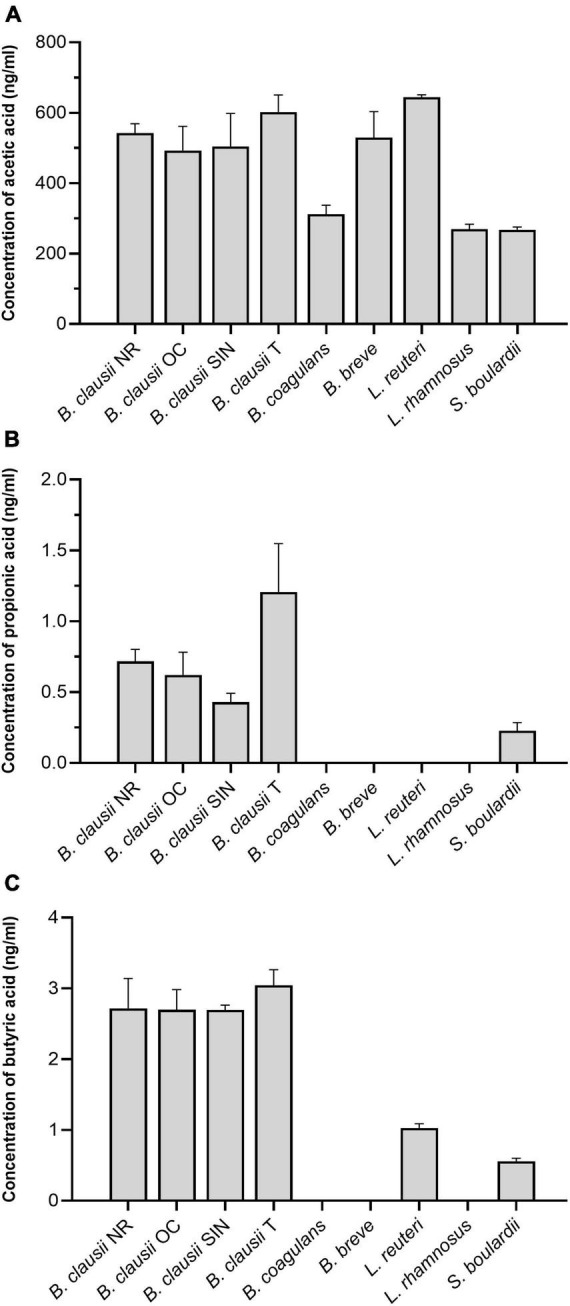
**(A)** Concentration of acetic acid (ng/mL) in the culture supernatants of *B. clausii* (NR, OC, SIN, and T), *B. coagulans*, *B. breve*, *L. reuteri*, *L. rhamnosus*, and *S. boulardii*. NR = *B. clausii* NR; OC = *B. clausii* OC; SIN = *B. clausii* SIN; T = *B. clausii* T; BC = *B. coagulans*; BB = *B. breve*; LRe = *L. reuteri*; LRh = *L. rhamnosus*; SB = *S. boulardii.* *P < 0.05, ^**^P < 0.01, ^***^P < 0.001, ^****^P < 0.0001. NR vs. BC^**^, NR vs. LRh^***^, NR vs. SB^***^, OC vs. BC*, OC vs. LRh^**^, OC vs. SB^**^, SIN vs. BC*, SIN vs. LRh^**^, SIN vs. SB^**^, T vs. BC^***^, T vs. LRh^****^, T vs. SB^****^, BC vs. BB^**^, BC vs. LRe^****^, BB vs. LRh^***^, BB vs. SB^***^, LRe vs. LRh^****^, LRe vs. SB^****^. **(B)** Concentration of propionic acid (ng/mL) in the culture supernatants of *B. clausii* (NR, OC, SIN, and T), *B. coagulans*, *B. breve*, *L. reuteri*, *L. rhamnosus*, and *S. boulardii.* NR vs. T*, OC vs. BC*, OC vs. BB*, OC vs. LRe*, OC vs. LRh*, SIN vs. T*, T vs. BC^****^, T vs. BB^****^, T vs. LRe^****^, T vs. LRh^****^, T vs. SB^***^. **(C)** Concentration of butyric acid (ng/mL) in the culture supernatants of *B. clausii* (NR, OC, SIN, and T), *B. coagulans*, *B. breve*, *L. reuteri*, *L. rhamnosus*, and *S. boulardii.* NR vs. BC^***^, NR vs. BB^***^, NR vs. LRe*, NR vs. LRh^***^, NR vs. SB^**^, OC vs. BC^****^, OC vs. BB^****^, OC vs. LRe^***^, OC vs. LRh^****^, OC vs. SB^****^, SIN vs. BC^****^, SIN vs. BB^****^, SIN vs. LRe^**^, SIN vs. LRh^****^, SIN vs. SB^***^, T vs. BC^****^, T vs. BB^****^, T vs. LRe^***^, T vs. LRh^****^, T vs. SB^****^.

Regarding acetic acid (Figure 2A), *B. clausii* T and *L. reuteri* resulted the highest producers among the tested strains, secreting 602.00 ± 54.15 ng/mL and 644.33 ± 7.15 ng/mL, respectively. No differences were highlighted among the four *B. clausii* strains. Secretion of acetic acid from *B. coagulans*, *L. rhamnosus*, and *S. boulardii* was significantly lower compared to *B. clausii* NR (BC: *P* = 0.0023; LRh and SB: *P* = 0.0003), OC (BC: *P* = 0.0215; LRh: *P* = 0.0031; SB: *P* = 0.0029), SIN (BC: *P* = 0.0132; LRh: *P* = 0.0019; SB: *P* = 0.0018), T (BC: *P* = 0.0002; LRh and SB: *P* < 0.0001), *B. breve* (BC: *P* = 0.0042; LRh and SB: *P* = 0.0006), and *L. reuteri* (*P* < 0.0001).

Propionic acid concentrations were found to be three orders of magnitude lower than acetic acid in the culture supernatants (Figure 2B). *B. coagulans*, *B. breve*, *L. reuteri*, and *L. rhamnosus* did not secrete propionic acid at all in our conditions. *B. clausii* T was able to produce the highest levels of propionic acid (1.21 ± 0.38 ng/mL), resulting significantly different from NR (*P* = 0.0374), SIN (*P* = 0.0112), and *S. boulardii* (*P* = 0.0007).

The levels of butyric acid were found to be slightly higher than those of propionic acid (Figure 2C). *B. coagulans*, *B. breve*, and *L. rhamnosus* were unable to secrete butyric acid, while all *B. clausii* strains showed a comparable secretion (NR: 2.72 ± 0.47 ng/mL; OC: 2.70 ± 0.31 ng/mL; SIN: 2.70 ± 0.06 ng/mL; T: 3.04 ± 0.25 ng/mL). A higher concentration of butyric acid was found in *B. clausii* NR, OC, SIN, and T culture supernatants compared to *L. reuteri* (NR: *P* = 0.0380; OC and T: *P* = 0.0002; SIN: *P* = 0.0012) and *S. boulardii* (NR: *P* = 0.0029; OC: *P* = 0.0001; SIN and T: *P* < 0.0001).

## Discussion

Short-chain fatty acids are well-known to play important roles in the promotion and maintenance of the health status and systemic homeostasis (Silva et al., 2020) and SCFA-producing probiotics have been proposed as possible implementation in the case of SCFA-deficiencies. Different SCFAs share some biological functions, but each SCFA also possesses peculiar properties and beneficial effects (Tan et al., 2014; Morrison and Preston, 2016; Riviere et al., 2016; Christiansen et al., 2018; Goswami et al., 2018; Liu et al., 2019, 2021; Okamoto et al., 2019; Silva et al., 2020; Langfeld et al., 2021; Gheorghe et al., 2022; O’Riordan et al., 2022). Therefore, the choice of a probiotic strain able to produce one or another SCFA should take into consideration the effects a particular acid displays on the host.

Different HPLC technologies have been used over years to quantify SCFAs (De Baere et al., 2013; Zheng et al., 2019; Chen et al., 2021), especially in studies where the intestinal microbiota was altered in association with various pathological conditions (Yamada et al., 2015; Garcia-Mantrana et al., 2018; Xue et al., 2019; Sowah et al., 2020; Soriano-Lerma et al., 2021). In our study, coupling tandem mass spectrometry to HPLC (HPLC-MS-MS) to quantify acetic, propionic, and butyric acids in microbial culture supernatants led to a very specific and sensitive detection, which is properly required when low analyte concentrations are present, as in the case of SCFAs (Marzo and Dal Bo, 2007; Leung and Fong, 2014).

The *in vitro* evaluation of SCFA secretion by probiotic strains can provide dissimilar results, even when the same microbial strain is tested, probably due to the culture conditions and media used for microbial growth (Asarat et al., 2015; LeBlanc et al., 2017). In fact, experimental protocols and environmental conditions influence the outcomes of analyses in terms of both quality and quantity of secreted SCFAs. For this reason, to guarantee a uniform microbial growth and obtain comparable results from the different tested strains, in this study a unique culture medium (i.e., BHIG) containing substantial concentrations of glucose and amino acids, which are the main essential substrates for SCFA synthesis, was selected and the same culture conditions were applied.

Among members of the *Bacillus* genus, *B. clausii* and *B. coagulans* have been used as probiotics for years considering the beneficial effects exerted in several gastrointestinal disorders (Lee et al., 2019; Shinde et al., 2020a,b). Since no evidence is present in the literature regarding their direct production of SCFAs, the present study highlights new metabolic features of these species. The ability of strains NR, OC, SIN, and T to secrete acetic, propionic, and butyric acids in our *in vitro* model suggests the potential of the *B. clausii* species to produce SCFAs. These compounds could contribute to the properties this species demonstrated as adjuvant treatment in several gastrointestinal dysfunctions (Ianiro et al., 2018; de Castro et al., 2019, 2020; Paparo et al., 2020; Plomer et al., 2020; Szajewska et al., 2020). *B. coagulans* strains are worldwide recognized as effective probiotics, and the role of *B. coagulans* SANK 70258 in ameliorating ulcerative colitis and leading the gut microbiota composition toward the enrichment in butyrate-producing bacteria has recently been shown (Sasaki et al., 2020). Herein, *B. coagulans* ATCC 7050 was proven to be able to secrete acetic acid, while propionic and butyric acids were not detected in its culture supernatant.

*Bifidobacterium* spp. have been demonstrated to confer many benefits to the human health, mainly when administered for pediatric pathologies, such as allergies, obesity, diarrhea, colic, and celiac disease (Bozzi Cionci et al., 2018). Different strains of *B. breve* are widely effective in preventing or ameliorating symptoms of several diseases, including Alzheimer’s disease (i.e., *B. breve* A1) and obesity-associated insulin sensitivity (i.e., *B. breve* BR03 and B632) by directly or indirectly modulating the local concentration of SCFAs (Kobayashi et al., 2017; Solito et al., 2021). In the present investigation, high concentrations of acetic acid were found in the culture supernatant of *B. breve* DSM 16604, which was never tested before for SCFA production. As expected, propionic and butyric acids were not detected, since the biosynthetic pathways for propionate and butyrate are not present in *Bifidobacterium* species (Ruiz-Aceituno et al., 2020).

Numerous lactobacilli are commonly administered as probiotics due to their beneficial properties (Zhang et al., 2018). Among lactobacilli, *L. reuteri* DSM 17938 is a well-characterized and largely commercialized probiotic microorganism, found to be suitable for the prevention and co-treatment of chronic constipation, colic, diarrhea, and gastroenteritis, especially in children (Gutiérrez-Castrellón et al., 2017; Kołodziej and Szajewska, 2019; Patro-Gołąb and Szajewska, 2019; Kubota et al., 2020). The ability of *L. reuteri* to produce SCFAs is a strain-dependent feature, as previously evidenced for *L. reuteri* NCIMB 11951, 701359, 701089, 702655, and 702656 (Kahouli et al., 2015). Herein, we demonstrated the ability of *L. reuteri* DSM 17938 to secrete large amounts of acetic acid and butyric acid to a lesser extent, thus suggesting a possible mechanism of action for reaching the health benefits associated to its administration. *L. rhamnosus*, whose persistence on the international market has lasted for more than 30 years due to its efficacy in managing several clinical conditions, is another bacterial species considered to have excellent probiotic properties (Capurso, 2019). *L. rhamnosus* strains were often demonstrated to be able to promote butyrogenesis and shape the gut microbiota with increased abundances of butyrate-producing bacteria (Lin et al., 2020). *L. rhamnosus* GG turned out to secrete acetic, propionic, and butyric acids in skim milk supplemented with prebiotics, as reported by Asarat et al. (2015), while another study showed the release of only propionic acid by this strain in MRS (LeBlanc et al., 2017). In this study, *L. rhamnosus* ATCC 53103 was able to secrete acetic acid in BHIG, but propionic and butyric acids were not detected in its culture supernatant. Our findings on *B. breve, L. reuteri*, and *L. rhamnosus* are in line with previous observations reporting *Bifidobacterium* and *Lactobacillus* species as mainly acetate producers (Cheng et al., 2021).

Several species of *Saccharomyces* are intrinsically able to determine an enrichment of SCFA-producing bacteria in the gut microbiota (Moré and Swidsinski, 2015; Offei et al., 2019), but only a few acidify the intestinal environment through the secretion of high levels of acetic acid themselves (Offei et al., 2019). Up to date, no information about secretion of propionic and butyric acid by *S. boulardii* is available in the literature. Although many efforts have been made on *S. cerevisiae* strains for enhancing the production of SCFAs by genetic engineering (Leber and Da Silva, 2014; Yu et al., 2016), a clear characterization of *S. boulardii* ability to secrete SCFAs is still lacking. *S. boulardii* CNCM I-745 was shown to release both acetic, propionic, and butyric acids in its culture supernatant, confirming its potential as regards SCFA metabolism.

Considering that the *in vitro* conditions used in this study for testing SCFA production are far from the actual environment and conditions found in the human intestine, further studies are needed to elucidate if the behaviors observed *in vitro* for the tested probiotic strains could also be observed *in vivo*. In fact, the presence of the gut microbiota, nutrients, pH, oxygen gradient, and many other factors could influence SCFA secretion by probiotics *in vivo*. Nevertheless, the *in vitro* analysis described in this study appears useful as a screening to evaluate the microbial potential to secrete SCFAs and help in the primary selection of promising SCFA-producing probiotic strains.

## Conclusion

The application of a novel sensitive HPLC-MS-MS protocol for the detection and quantification of SCFAs allowed us to establish that all the tested probiotic strains are able to actively secrete acetic acid and a part of them all the three main short-chain fatty acids *in vitro*. Although our study cannot exclude a different microbial behavior *in vivo* or in other *in vitro* conditions, we recommend that the production of SCFAs should be taken into consideration as key feature when next generation probiotics and psychobiotics are evaluated for their potential clinical effectiveness. An in-depth characterization of strains contained in probiotic formulations as regards SCFA secretion could be a novel aspect to consider in the probiotic research and contribute to the spread of more targeted and personalized bacteriotherapy strategies to promote human health and manage diseases.

## Data availability statement

The raw data supporting the conclusions of this article will be made available by the authors, without undue reservation.

## Author contributions

RZ, AS, and EG: conception and design of the study, validation, and formal analysis. MC, AB, SC, RZ, AS, and EG: methodology. MC, AB, SC, DM, AP, MM, and FC: investigation. MC and AB: writing—original draft preparation. MC, AB, SC, DM, AP, MM, FC, RZ, AS, and EG: writing—review and editing. EG: supervision. All authors read and agreed to the published version of the manuscript.
