# Supplementary material for: HPLC-MS-MS quantification of short-chain fatty acids actively secreted by probiotic strains
Source: Front Microbiol. 2023 Mar 3;14:1124144. doi: 10.3389/fmicb.2023.1124144 (PMC10020375; doi:10.3389/fmicb.2023.1124144)
Supplement: Supplementary file 1 [file Table_1.DOCX]

**Tab. S1** MS operative parameters

| **Analyte** | **SRM Transition** | **CE^1^ (V)** | **CxP^2^ (V)** |
| --- | --- | --- | --- |
| Acetic acid | 194.1 🡪 136.9 (Q) | -24.7 | -3.5 |
|  | 194.1 🡪 152.1 (q) | -17.1 | -4.0 |
|  | 194.1 🡪 178.0 (q) | -18.0 | -3.2 |
| ^13^C_2_-acetic acid | 196.1 🡪 137.2 (Q) | -24.7 | -3.5 |
|  | 196.1 🡪 152.0 (q) | -17.1 | -4.0 |
|  | 196.1 🡪 178.0 (q) | -18.0 | -3.2 |
| Propionic acid | 208.1 🡪 137.1 (q) | -26.0 | -3.6 |
|  | 208.1 🡪 152.2 (q) | -26.0 | -3.6 |
|  | 208.1 🡪 165.0 (Q) | -17.5 | -4.7 |
| ^13^C_2_-propionic acid | 210.1 🡪 137.1 (q) | -26.0 | -3.6 |
|  | 210.1 🡪 152.1 (q) | -26.0 | -3.6 |
|  | 210.1 🡪 167.0 (Q) | -17.5 | -4.7 |
| Butyric acid | 222.1 🡪 136.9 (q) | -26.0 | -11.0 |
|  | 222.1 🡪 152.1 (Q) | -20.0 | -4.4 |
|  | 222.1 🡪 178.8 (q) | -9.0 | -4.9 |

^1^ collision energy

^2^ collision exit potential
